# The association between neighbourhood characteristics and physical victimisation in men and women with mental disorders

**DOI:** 10.1192/bjo.2020.52

**Published:** 2020-07-16

**Authors:** Vishal Bhavsar, Jyoti Sanyal, Rashmi Patel, Hitesh Shetty, Sumithra Velupillai, Robert Stewart, Matthew Broadbent, James H. MacCabe, Jayati Das-Munshi, Louise M. Howard

**Affiliations:** Section of Women's Mental Health, Institute of Psychiatry, Psychology & Neuroscience, King's College London, UK; Clinical Informatics, BRC Nucleus, South London and Maudsley NHS Foundation Trust, UK; Department of Psychosis Studies, Institute of Psychiatry, Psychology & Neuroscience, King's College London, UK; Clinical Informatics, BRC Nucleus, South London and Maudsley NHS Foundation Trust, UK; King's College London, UK; BRC Nucleus, South London and Maudsley NHS Foundation Trust, UK; Clinical Informatics, BRC Nucleus, South London and Maudsley NHS Foundation Trust, UK; Department of Psychosis Studies, King's College London, UK; Department of Health Services and Population Research, King's College London, UK; Section of Women's Mental Health, Institute of Psychiatry, Psychology & Neuroscience, King's College London, UK

**Keywords:** Natural language processing, violence, neighbourhood characteristics, electronic health records, data linkage

## Abstract

**Background:**

How neighbourhood characteristics affect the physical safety of people with mental illness is unclear.

**Aims:**

To examine neighbourhood effects on physical victimisation towards people using mental health services.

**Method:**

We developed and evaluated a machine-learning-derived free-text-based natural language processing (NLP) algorithm to ascertain clinical text referring to physical victimisation. This was applied to records on all patients attending National Health Service mental health services in Southeast London. Sociodemographic and clinical data, and diagnostic information on use of acute hospital care (from Hospital Episode Statistics, linked to Clinical Record Interactive Search), were collected in this group, defined as ‘cases’ and concurrently sampled controls. Multilevel logistic regression models estimated associations (odds ratios, ORs) between neighbourhood-level fragmentation, crime, income deprivation, and population density and physical victimisation.

**Results:**

Based on a human-rated gold standard, the NLP algorithm had a positive predictive value of 0.92 and sensitivity of 0.98 for (clinically recorded) physical victimisation. A 1 s.d. increase in neighbourhood crime was accompanied by a 7% increase in odds of physical victimisation in women and an 13% increase in men (adjusted OR (aOR) for women: 1.07, 95% CI 1.01–1.14, aOR for men: 1.13, 95% CI 1.06–1.21, *P* for gender interaction, 0.218). Although small, adjusted associations for neighbourhood fragmentation appeared greater in magnitude for women (aOR = 1.05, 95% CI 1.01–1.11) than men, where this association was not statistically significant (aOR = 1.00, 95% CI 0.95–1.04, *P* for gender interaction, 0.096). Neighbourhood income deprivation was associated with victimisation in men and women with similar magnitudes of association.

**Conclusions:**

Neighbourhood factors influencing safety, as well as individual characteristics including gender, may be relevant to understanding pathways to physical victimisation towards people with mental illness.

## Background

Physical violence is a common and preventable cause of morbidity and mortality in people with mental illness^1^ and has a negative impact on quality of life and treatment response.^2^ A 2016 systematic review of 30 studies found a strong association of severe mental illness with victimisation in both men and women, in comparison with the general population.^3^ Large register-based epidemiological studies in the USA,^4^ Sweden^5^ and Denmark^6^ confirm the association between mental disorders and subsequent experience of violent crime.

## Victimisation

The World Health Organization ecological framework emphasises neighbourhood and community context, alongside victim and perpetrator characteristics, in the occurrence of violence.^7^ Victimisation displays important gender differences; in the general population, men experience greater physical victimisation than women, whereas violence in domestic settings affects more women than men.^8^ In surveys of the general population, experiencing physical violence as a victim (physical victimisation) is associated with individual characteristics such as younger age, minority ethnicity, single marital status and use of drugs and alcohol, but also with neighbourhood deprivation,^9^ and residing in areas with greater population density.^10^ Risk of victimisation is influenced by the availability of settings where violence may more easily occur, the likelihood of interacting with a possible perpetrator and the local presence of risk factors for violence. The level of neighbourhood crime has therefore been evaluated as a risk factor for victimisation in the general population.^11^

## Neighbourhood characteristics

Neighbourhood characteristics are also associated with mental illness. Neighbourhood deprivation is associated with occurrence of mental illness, including psychosis^12^ and depression.^13^ It has also been suggested that public mental health may be improved by nurturing neighbourhood social networks and local reserves of material resources, support, and trusting relationships accessible by people when they experience stress, adversity and disadvantage.^14^ A construct that captures these aspects is neighbourhood fragmentation, defined as the degree of social disorganisation, residential turnover and relationship breakdown in a neighbourhood. Fragmented neighbourhoods may display greater occurrence of severe mental illness,^15,16^ after accounting for individual characteristics.^17^

## Aims

Identifying risk factors for physical victimisation in mental illness may provide avenues for developing effective interventions. However, there has been limited examination of neighbourhood characteristics as influences on physical victimisation in people with mental illness. Previous epidemiological investigations have typically used participant interviews, routine data and surveys to ascertain physical victimisation, with each method introducing possible differential under-ascertainment of all physical victimisation affecting the population.^18^ Physical victimisation identified by clinical services, for example during patient assessments and history-taking, could reflect incidents not collected through other sources, and strengthen evidence for interventions to improve patient safety. We examined neighbourhood effects on physical victimisation towards people using mental health services, testing gender-specific associations with neighbourhood characteristics within a multilevel conceptual framework.

## Method

### Data source

The study was carried out in accordance with the RECORD statement^19^ (see Supplementary Checklist, available at https://doi.org/10.1192/bjo.2020.52). We did a case–control study using two linked databases, one containing mental health records and another containing hospital admissions data. We used a natural language processing (NLP) algorithm, developed and evaluated for the purposes of this study to define ‘cases’ and controls.

Algorithm development and evaluation is described further in the Supplementary Methods. The first data source for this study was the South London and Maudsley (SLaM) Biomedical Research Centre Clinical Record Interactive Search (CRIS) system, comprising complete de-identified electronic health records from the comprehensive National Health Service (NHS) mental healthcare provider in South East London, offering services for residents of the London boroughs of Lambeth, Southwark, Lewisham, and Croydon (comprising a total population of around 1.2 million^20^).

**Fig. 1:**
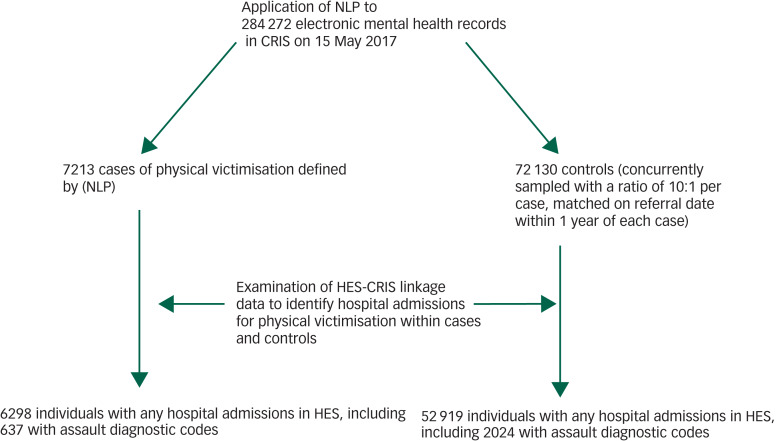
Flow diagram to demonstrate linked databases included in this study.

NLP, natural language processing; HES, Hospital Episode Statistics; CRIS, Clinical Record Interactive Search.

The register included data from clinical free text entered by clinicians, documents of clinical correspondence, structured fields for scales/questionnaires and sociodemographic data, since 2006 for all SLaM services. The CRIS database has been linked to the Hospital Episode Statistics (HES) Admitted Patient Care (APC) (HES-APC) data-set, a comprehensive record of all NHS hospital in-patient admissions in England since 2014.^21^ These linked data were used to provide information on use of in-patient medical care for victimisation. Alongside the NLP-derived definition of cases (described in the methodological supplement) we also inspected associations with a case definition based on HES admission for assault, drawn from linkage with the HES-APC data-set. The aim of using hospital admission cases was to check if associations with the NLP case definition were consistent in their magnitude/direction, when using a different case definition (incorporating HES data). See Fig. 1 for a flow diagram summarising the linked databases and flow of participants through the study.

### Consent and ethics statement

All individual data was anonymous. Therefore, informed consent from participants was not sought. The authors assert that all procedures contributing to this work comply with the ethical standards of the relevant national and institutional committees on human experimentation and with the Helsinki Declaration of 1975, as revised in 2008. Ethical approval for CRIS was granted by the Oxford REC, reference 18/SC/0372.

### Identification of physical victimisation cases and controls

The algorithm was applied to CRIS (15 May 2017; 284 272 individual patient records). The algorithm generated a binary variable for each participant, for any clinical documentation of physical victimisation, occurring at any time in a person's lifetime (and recorded in clinical records from 2006). This variable was used to ascertain people with a very high probability of lifetime physical victimisation, who were defined as cases. For each case, ten controls, defined as individuals who were not identified with physical victimisation by the NLP algorithm, with referral dates falling within 1 year of the corresponding case, were also randomly sampled. This was in order to optimise power to detect possibly small associations. Linkage of CRIS with medical in-patient data from HES data for England and Wales had been previously established and is described elsewhere.^20^ We used this linkage to examine the case definition for our analyses. To identify admissions for victimisation, we used these linked data to identify the presence of at least one hospital admission involving ICD-10 codes^22^ for assault, which were: X85–99, Y00–Y04 and Y08–Y09. The diagnostic codes included in the HES definition of hospital admission for physical victimisation for this study are displayed in Supplementary Table 2.

### Neighbourhood characteristics

Addresses at which cases and controls were residing at the time of referral to mental health services were used to derive information on neighbourhood characteristics. All neighbourhood characteristics were taken at the geographic level of the 2011 lower super output areas (LSOA), which are small geographic units enclosing an average of 1500 residents. Neighbourhood crime was measured using the Index for Multiple Deprivation crime domain for 2010.^23^ Neighbourhood fragmentation was measured using the Congdon Index for neighbourhood fragmentation,^24^ a composite indicator based on 2011 data on population turnover, percentage of privately rented households, single person households and unmarried people. Neighbourhood socioeconomic status was measured using the income deprivation domain of the Index for Multiple Deprivation 2010. We also assessed the impact of including overall neighbourhood deprivation, rather than neighbourhood income deprivation, on estimates. Population density was measured using persons per hectare, based on census data from 2011. All neighbourhood characteristics data were positively scaled (i.e. higher scores indicating greater crime, fragmentation and income deprivation, respectively), and *z*-standardised for ease of interpretation of estimates, to reflect a mean of 0 and a s.d. of 1. The measurement of other analysed variables is described in the methodological supplement.

### Analysis

Analyses were carried out in Stata 14. We described counts, proportions, and χ^2^-tests of physical victimisation with age at referral (categorised for descriptive purposes into age groups 0–15, 16–24, 25–35, 36–50, ≥51), gender, ethnic group, marital status, primary diagnosis, the presence of comorbid drug or alcohol use disorders, and any record for hospital admission for physical victimisation in HES. Crude associations of physical victimisation with neighbourhood characteristics (neighbourhood fragmentation, neighbourhood crime, neighbourhood income deprivation, and population density) were described by comparing medians, means and *t*-tests. The correspondence of NLP-derived physical victimisation with hospital admission data was assessed by calculating the proportion of the case groups with at least one hospital admission for physical victimisation, and by reporting this proportion within strata of covariates included in this study, including neighbourhood characteristics (presented in Supplementary Table 3).

We then modelled association between neighbourhood characteristics (neighbourhood fragmentation, neighbourhood crime, neighbourhood income deprivation and neighbourhood population density) and physical victimisation, based on the NLP algorithm. Because all neighbourhood characteristics were *z*-standardised (that is, set to have a mean of 0 and s.d. of 1), all logistic regression model coefficients for neighbourhood characteristics reflected the relative change in odds of physical victimisation for an increase of 1 s.d. in the neighbourhood characteristic. To evaluate collinearity affecting the stability and precision of model estimates, crude correlations among neighbourhood characteristics were evaluated using pairwise correlation coefficients and presented in a matrix (see Supplementary Table 4). Continuous variables were not entered in models together if the pairwise correlation between the two variables was greater than 0.7. All continuous covariates were assessed for goodness of fit as linear, quadratic and categorical indicator terms (in quintiles) using the Bayes Information Criteria.

In order to account for the clustering of neighbourhood characteristics within individuals residing in the same neighbourhoods, all models included a neighbourhood (LSOA)-level random effect, using the melogit command in Stata, and were estimated using robust standard errors. In primary analyses, age, gender, a multiplicative interaction term for gender, marital status and ethnic group were included in final models as forced covariates. Diagnostic group, and comorbid drug or alcohol use disorders, were evaluated for inclusion in final models, so as to maintain parsimony of the model. These covariates were included only if their inclusion changed the estimate by greater than 10% compared with the crude association.^25^

Having identified covariates for inclusion in the final model, adjusted estimates were reported by estimating random effects logistic regression models including (a) each neighbourhood characteristic without other covariates, (b) adding only individual-level covariates, (c) by adding only the other neighbourhood covariates, and (d) including all variables in order to arrive at a fully adjusted estimate. All models employed linear combinations estimating gender-specific associations between neighbourhood characteristics and physical victimisation, and we reported model estimates associations for women, and post-estimation fitted estimates for the association in men. Finally, we estimated absolute risk differences for a difference in 1 s.d. from the mean, for neighbourhood characteristics by gender, based on final model estimates. Missing data on all variables included in final models were described by case–control status, and missing data proportions compared.

## Results

### Descriptive results

Based on a human-rated gold standard, the NLP algorithm had a positive predictive value of 0.92 and sensitivity of 0.98 for (clinically recorded) physical victimisation. We identified 7213 users of mental health services with a history of physical victimisation based on the NLP algorithm described, giving an overall prevalence of 2.5%. Comparison of this group with 72 130 concurrently sampled controls, without recorded physical victimisation, indicated association of physical victimisation with younger age, male gender, Black and mixed, ethnic group, and single and divorced marital status (all *P* < 0.001, see Table 1). Individuals with physical victimisation were most commonly diagnosed with psychotic disorders (20.4%) and mood disorders (16.3%). Based on HES-linkage, 8.8% of those identified as cases through NLP experienced at least one hospital admission for physical victimisation (Table 1).

**Table 1:** Descriptive data on cases, with natural language processing-derived physical victimisation in health records, and controls, with column percentages for each covariate

	Control group	Case group	Total	χ^2^	*P*
Age, years					
0–15, *n*	11 261	1292	12 554		
0–15, %	15.61	17.91	15.82		
16–24, *n*	11 780	1534	13 314		
16–24, %	16.33	21.27	16.78		
25–35, *n*	15 769	1796	17 565		
25–35, %	21.86	24.90	22.14		
36–50, *n*	15 840	1709	17 549		
36–50, %	21.96	23.69	22.12		
≥51, *n*	17 410	878	18 288		
≥51, %	24.14	12.17	23.05	560.60	<0.001
Missing, *n*	70	3	73		
Missing, %	0.10	0.04	0.09		
Gender					
Women, *n*	36 510	3036	39 546		
Women, %	50.62	42.09	49.84		
Men, *n*	35 600	4177	39 777		
Men, %	49.36	57.91	50.13	191.30	<0.001
Missing, *n*	20	0	20		
Missing, %	0.03	0.00	0.03		
Ethnic group					
White, *n*	37 156	3722	40 878		
White, %	51.51	51.60	51.52		
Mixed, *n*	1751	337	2088		
Mixed, %	2.43	4.67	2.63		
Asian, *n*	2665	296	2961		
Asian, %	3.69	4.10	3.73		
Black, *n*	10 483	2162	12 645		
Black, %	14.53	29.97	15.94		
Other, *n*	4453	416	4869		
Other, %	6.17	5.77	6.14	723.36	<0.001
Missing, *n*	15 622	280	15 902		
Missing, %	21.66	3.88	20.04		
Marital status					
Single, *n*	32 499	4906	37 405		
Single, %	45.06	68.02	47.14		
Married or cohabiting, *n*	10 320	764	11 084		
Married or cohabiting, %	14.31	10.59	13.97		
Divorced or separated, *n*	4143	571	4714		
Divorced or separated, %	5.74	7.92	5.94		
Widowed, *n*	3692	190	3882		
Widowed, %	5.12	2.63	4.89	501.14	<0.001
Missing, *n*	21 476	782	22 258		
Missing, %	29.77	10.84	28.05		
Primary ICD-10 diagnosis					
F0–9: Organic mental disorders, *n*	4474	222	4696		
F0–9: Organic mental disorders, %	6.20	3.08	5.92		
F10–19: Mental and behavioural disorders due to psychoactive substance use, *n*	5647	523	6170		
F10–19: Mental and behavioural disorders due to psychoactive substance use, %	7.83	7.25	7.78		
F20–29: Schizophrenia, schizotypal and delusional disorders, *n*	3458	1472	4930		
F20–29: Schizophrenia, schizotypal and delusional disorders, %	4.79	20.41	6.21		
F30–39: Mood (affective) disorders, *n*	8647	1177	9824		
F30–39: Mood (affective) disorders, %	11.99	16.32	12.38		
F40–49: Neurotic, stress-related and somatoform disorders, *n*	6541	619	7160		
F40–49: Neurotic, stress-related and somatoform disorders, %	9.07	8.58	9.02		
F50–59: Behavioural syndromes associated with physiological disturbances and physical factors, *n*	1944	65	2009		
F50–59: Behavioural syndromes associated with physiological disturbances and physical factors, %	2.70	0.90	2.53		
F60–69: Disorders of adult personality and behaviour, *n*	898	237	1135		
F60–69: Disorders of adult personality and behaviour, %	1.24	3.29	1.43		
F70–79: Learning disability, *n*	758	156	914		
F70–79: Learning disability, %	1.05	2.16	1.15		
F80–89: Disorders of psychological development, *n*	1361	149	1510		
F80–89: Disorders of psychological development, %	1.89	2.07	1.90		
F90–98: Behavioural and emotional disorders with onset usually occurring in childhood and adolescence, *n*	3171	403	3574		
F90–98: Behavioural and emotional disorders with onset usually occurring in childhood and adolescence, %	4.40	5.59	4.50		
F99: Unspecified mental disorder, *n*	10 075	760	10 835		
F99: Unspecified mental disorder, %	13.97	10.54	13.66		
No axis 1 diagnosis, *n*	3244	338	3582		
No axis 1 diagnosis, %	4.50	4.69	4.51		
G: Diseases of the nervous system, *n*	203	14	217		
G: Diseases of the nervous system, %	0.28	0.19	0.27		
A-E, H-Q: Other illness codes, *n*	238	12	250		
A-E, H-Q: Other illness codes, %	0.33	0.17	0.32		
R: Symptoms, signs and abnormal clinical and laboratory findings, not elsewhere classified, *n*	401	11	412		
R: Symptoms, signs and abnormal clinical and laboratory findings, not elsewhere classified, %	0.56	0.15	0.52		
S-Y: Injury, poisoning and external causes, *n*	96	12	108		
S-Y: Injury, poisoning and external causes, %	0.13	0.17	0.14		
Z: Factors influencing health status and contact with health services, *n*	11 868	850	12 718	3 × 10^3^	<0.001
Z: Factors influencing health status and contact with health services, %	16.45	11.78	16.03		
Missing, *n*	9106	193	9299		
Missing, %	12.62	2.68	11.72		
Any comorbid diagnosis of drug or alcohol use disorder					
No, *n*	71 665	7111	78 766		
No, %	99.35	98.59	99.27		
Yes, *n*	475	102	577		
Yes, %	0.66	1.41	0.73	51.85	<0.001
HES assault admission					
No, *n*	70 151	6576	76 727		
No, %	97.26	91.17	96.70		
Yes, *n*	1979	637	2616		
Yes, %	2.74	8.83	3.30	762.1659	<0.001
Total, *n*	72 130	7213	79 343		

HES, Hospital Episode Statistics.

Case status was associated with greater neighbourhood fragmentation, higher neighbourhood crime, and higher neighbourhood income deprivation, and greater population density, compared with controls (all *P* < 0.001, Table 2). NLP-defined cases who also experienced hospital admission for physical victimisation were more commonly from younger age groups, men, of single marital status, diagnosed with comorbid alcohol and drug use disorders, and resided in neighbourhoods with lower neighbourhood income deprivation (Supplementary Table 3).

**Table 2:** Neighbourhood characteristics in cases and controls^a^

	Control group	Case group	*t*-test *P*
Neighbourhood fragmentation, mean (s.d.)	0.00 (1.00)	0.08 (0.91)	<0.001
Neighbourhood fragmentation, median (IQR)	0.02 (1.30)	0.06 (1.16)	
Neighbourhood crime, mean (s.d.)	0.27 (0.81)	0.41 (0.74)	<0.001
Neighbourhood crime, median (IQR)	0.36 (0.99)	0.47 (0.85)	
Neighbourhood income deprivation, mean (s.d.)	0.09 (0.89)	0.29 (0.82)	<0.001
Neighbourhood income deprivation, median (IQR)	0.08 (1.33)	0.33 (1.20)	
Neighbourhood population density, mean (s.d.))	0.28 (0.97)	0.41 (0.91)	<0.001
Neighbourhood population density, median (IQR)	0.20 (1.25)	0.34 (1.18)	

IQR, interquartile range.

a. All neighbourhood characteristics were positively scaled, that is, a higher score indicates greater fragmentation, crime, income deprivation and population density, respectively.

Pairwise correlations all suggested low or moderate correlation among neighbourhood fragmentation, neighbourhood crime, neighbourhood income deprivation and neighbourhood population density (see Supplementary Table 4). Associations of each covariate with physical victimisation did not vary when using a more restrictive outcome definition, based on the presence of both the NLP case definition and hospital admission for physical victimisation (see Supplementary Table 5).

### The associations of neighbourhood characteristics with physical victimisation

Table 3 presents partially and fully adjusted model estimates for women and for men, all based on 44 475 individuals with complete data on modelled variables, clustered in 2794 LSOA. For women, neighbourhood fragmentation was associated with 11% higher odds of physical victimisation, which attenuated to 5% (odds ratio (OR) = 1.05, 95% CI 1.01–1.11) on adjustments. Neighbourhood crime was associated with 30% higher odds of physical victimisation in women, brought down to 7% after adjustments (OR = 1.07, 95% CI 1.01–1.14, see Table 4).

**Table 3:** Model estimates for association of neighbourhood characteristics, based on 44 475 records with complete data, clustered in 2794 neighbourhoods (lower super output areas)

	Unadjusted^a^	Individual-adjusted^b^	Area-adjusted^c^	Both individual- and area-adjusted^d^	Absolute risk difference, %^e^
Women					
Neighbourhood fragmentation	1.11 (1.06 to 1.16)	1.06 (1.02 to 1.11)	1.07 (1.02 to 1.12)	1.05 (1.01 to 1.11)	0.51 (0.02 to 1.00)
Neighbourhood crime	1.30 (1.24 to 1.38)	1.19 (1.13 to 1.26)	1.13 (1.06 to 1.21)	1.07 (1.01 to 1.14)	0.59 (−0.06 to 1.23)
Neighbourhood income deprivation	1.29 (1.24 to 1.34)	1.18 (1.13 to 1.24)	1.20 (1.14 to 1.27)	1.14 (1.08 to 1.21)	1.26 (0.72 to 1.81)
Neighbourhood population density	1.17 (1.12 to 1.22)	1.08 (1.03 to 1.12)	1.05 (1.00 to 1.11)	0.99 (0.94 to 1.04)	−0.09 (−0.56 to 0.39)
Men					
Neighbourhood fragmentation	1.06 (1.01 to 1.11)	1.01 (0.97 to 1.05)	1.03 (0.98 to 1.08)	1.00 (0.95 to 1.04)^f^	−0.02 (−0.51 to 0.48)
Neighbourhood crime	1.28 (1.21 to 1.34)	1.18 (1.12 to 1.24)	1.19 (1.11 to 1.28)	1.13 (1.06 to 1.21)^g^	1.36 (0.67 to 2.04)
Neighbourhood income deprivation	1.21 (1.16 to 1.26)	1.12 (1.08 to 1.17)	1.15 (1.09 to 1.21)	1.10 (1.04 to 1.16)^h^	1.01 (0.44 to 1.59)
Neighbourhood population density	1.10 (1.06 to 1.15)	1.03 (0.99 to 1.08)	1.00 (0.95 to 1.05)	0.97 (0.92 to 1.01)^i^	−0.38 (−0.09 to 0.13)

a. The intraclass correlation coefficient (%) for the empty model, before inclusion of explanatory variables, was 2.7 (95% CI 2.0–3.8).

b. Adjusted for age, ethnic group, marital status, primary diagnosis and comorbid drug or alcohol use disorder.

c. Adjusted for the other neighbourhood characteristics.

d. Adjusted for age, ethnic group, marital status, primary diagnosis and comorbid drug or alcohol use disorder, and all other neighbourhood characteristics.

e. Based on the fully adjusted model, comparing absolute risks for a 1 s.d. increase in each neighbourhood characteristic.

f. *P* for gender by neighbourhood fragmentation interaction: 0.096.

g. *P* for gender by neighbourhood crime interaction: 0.218.

h. *P* for gender by neighbourhood income deprivation interaction: 0.257.

i. *P* for interaction between gender and neighbourhood population density: 0.448.

**Table 4:** All model estimates based on 44 475 records with complete data, for the association, in the form of odds ratios (ORs with 95% CIs) of neighbourhood characteristics with physical victimisation

	OR	Lower 95% CI	Upper 95% CI
Neighbourhood characteristics			
Neighbourhood fragmentation for women^a^	1.05	1.01	1.11
Neighbourhood fragmentation for men^b^	1.00	0.95	1.04
Neighbourhood crime for women^c^	1.07	1.01	1.14
Neighbourhood crime for men^d^	1.13	1.06	1.21
Neighbourhood income deprivation for women^e^	1.14	1.08	1.21
Neighbourhood income deprivation for men^f^	1.10	1.04	1.16
Neighbourhood population density for women^g^	0.99	0.94	1.04
Neighbourhood population density for men^h^	0.97	0.92	1.01
Lower super output area-level random effect	0.01	0.00	0.07
*Individual characteristics*			
Age^i^	0.98	0.98	0.98
Gender			
Women	Reference	–	–
Men	1.28	1.19	1.38
Ethnic group			
White	Ref		
Mixed	1.32	1.15	1.52
Asian	0.95	0.82	1.09
Black	1.37	1.27	1.47
Other	0.72	0.64	0.82
Marital status			
Single	Reference	–	–
Married or cohabiting	0.77	0.71	0.85
Divorced or separated	1.15	1.03	1.29
Widowed	0.83	0.69	1.01
Diagnostic group			
F0–9: Organic mental disorders	Reference	–	–
F10–19: Mental and behavioural disorders due to psychoactive substance use	0.79	0.65	0.95
F20–29: Schizophrenia, schizotypal and delusional disorders	3.82	3.23	4.53
F30–39: Mood (affective) disorders	1.56	1.32	1.85
F40–49: Neurotic, stress-related and somatoform disorders	0.94	0.78	1.13
F50–59: Behavioural syndromes associated with physiological disturbances	0.35	0.25	0.48
F60–69: Disorders of adult personality and behaviour	2.58	2.05	3.26
F70–79: Learning disability	1.78	1.36	2.33
F80–89: Disorders of psychological development	0.68	0.51	0.90
F90–98: Behavioural and emotional disorders with onset	0.73	0.58	0.91
F99: Unspecified mental disorder	0.89	0.74	1.07
No Axis I diagnosis	0.64	0.51	0.81
G: Diseases of the nervous system	1.42	0.75	2.68
A–E, H–Q: Other illness codes	0.83	0.45	1.54
R: Symptoms, signs and abnormal clinical and laboratory findings	0.38	0.19	0.76
S-Y: Injury, poisoning and external causes	1.43	0.63	3.26
Z: Factors influencing health status and contact with health services	0.92	0.76	1.10
Any comorbid diagnosis of drug or alcohol use disorders			
No	Reference	–	–
Yes	2.01	1.53	2.65

a. A squared term for neighbourhood fragmentation was included based on goodness of fit in crude models for physical victimisation, and estimated at 0.99 (95% CI 0.97–1.01).

b. *P* for gender by neighbourhood fragmentation interaction: 0.096.

c. Neighbourhood crime was taken from the crime deprivation domain of the Index for Multiple Deprivation 2011.

d. *P* for gender by neighbourhood crime interaction: 0.218.

e. Neighbourhood income deprivation was taken from the income domain of the Index for Multiple Deprivation 2011.

f. *P* for gender by neighbourhood income deprivation interaction: 0.257.

g. Population density in people per square kilometre.

h. *P* for interaction between gender and neighbourhood population density: 0.448.

i. Age was included as a linear term in years, based on goodness of fit.

Before adjustments, greater neighbourhood income deprivation was associated with 29% higher odds of physical victimisation in women, which was attenuated to 14% after adjustment for individual and neighbourhood characteristics (OR = 1.14, 95% CI 1.08–2.21). Higher neighbourhood population density was associated with a 17% greater odds of victimisation in women, however, this was substantially attenuated on adjustment (fully adjusted OR = 0.99, 95% CI 0.94, 1.04).

In men, there was a null association of neighbourhood fragmentation with physical victimisation after all adjustments (OR = 1.00, 95% CI 0.95–1.04). There was a slightly higher point estimate for association of neighbourhood crime with physical victimisation in men (OR = 1.13, 95% CI 1.06–1.21). Neighbourhood income deprivation was associated with a 10% increase in the odds of victimisation in men after adjustments (OR = 1.10, 95% CI 1.04–1.16). The fully adjusted association of neighbourhood population density and victimisation in men was close to null (OR = 0.97, 95% CI 0.92–1.01).

Our aims focused on associations between neighbourhood characteristics with victimisation in men and women. Therefore, we did not produce estimates pooled across men and women in the primary analysis but report these in Supplementary Table 6. Estimates were unchanged when we included neighbourhood deprivation, rather than neighbourhood income deprivation in final models. Absolute risk differences ranged from −0.38% (95% CI −0.09 to 0.13) for neighbourhood population density in men, to 1.36% (95% CI 0.67 to 2.04), for neighbourhood crime in men.

### Covariate model estimates

Among individual-level covariates, statistical associations with greater odds of physical victimisation were evident in final models for younger age, male gender, both mixed and Black ethnic groups (compared with the White reference group), divorced or separated marital status (compared with the single reference group), psychotic, mood, personality, and intellectual disability diagnostic groups (compared with the organic syndromes reference group), and the presence of a comorbid drug and alcohol use disorders (see Table 4). Married or cohabiting marital status was associated with a statistically lower odds of victimisation compared with single marital status.

### Missing data

Complete data was included in models from 44 475 individuals. Age and gender were missing in less than 1% of both the case and control groups (Supplementary Table 7). The case group had lower proportions of missing data than the controls for ethnic group, marital status and diagnosis. Missing data on neighbourhood fragmentation was between 11% and 15% for both the case and control groups, and between 5% and 7% for other neighbourhood characteristics. Crude associations between case–control status and neighbourhood within groups with missing data on each covariate were between 0.98 and 1.22, consistent with final estimates in this study.

## Discussion

### Summary of findings

We present the first, to our knowledge, multilevel examination of neighbourhood associations with physical victimisation in people with mental illness, ascertained through NLP. We found evidence for association between neighbourhood fragmentation and physical victimisation among women, but not men. In contrast, neighbourhood crime and neighbourhood income deprivation, remained associated with physical victimisation in both women and men, after accounting for potential confounders, while crude associations of neighbourhood population density with victimisation were completely attenuated after covariate adjustment, in both men and women. In our final model, physical victimisation in people using mental health services was also associated with younger age at referral, male gender, divorced/separated marital status, Black ethnic group, mixed ethnic group, diagnosis of psychotic, mood, and personality disorders, and comorbid drug and alcohol use disorders.^8^

### Explanation of findings

Our results suggest that although neighbourhood crime may influence physical victimisation in both men and women (after accounting for individual-level factors and neighbourhood income deprivation and population density), fragmentation is associated with physical victimisation in women, but not men, consistent with a small number of general population studies.^26–29^ Fragmented neighbourhoods are considered to offer more limited support structures at times of stress, need and privation, including access to third-sector support services. They may also offer fewer opportunities for meaningful activity, coping, safe physical activity and safe routines, which may all be necessary for maintaining personal safety. Neighbourhoods with greater levels of crime, antisocial behaviour, and rule breaking may also contain greater prevalence of perpetrators liable to commit crimes, including crimes towards people with vulnerabilities. It is possible that neighbourhood patterning of violent behaviour is one explanation for associations reported in this paper.

This violence could be more likely to occur outside the home, consistent with the gender differences observed in this study. On the other hand, violence against women with mental illness could be specifically driven by factors influencing the ability to access support, help, advocacy and resources in the wider community, partly reflected in the neighbourhood fragmentation variable investigated in this study. This is consistent with some literature on gender differences in neighbourhood associations with violence in the general population. In a study focusing specifically on dating violence, rather than any physical violence, Jain et al^27^ found that neighbourhood factors (concentrated poverty, perceived violence, collective efficacy) were more strongly associated with physical victimisation in men, than women. Cunradi and others^30^ examined the association of neighbourhood poverty with intimate partner violence, finding that statistical evidence remained after adjusting for income, marital status, number of children, educational attainment and socioeconomic status of both the victim and perpetrator. Although we were not able to distinguish between domestic and other types of violence in this study, it is possible that the observed neighbourhood differences between genders was related to neighbourhood factors being relevant to different types of physical victimisation.

In the general population, most violence occurs within the home, and in the context of intimate and family relationships. De Mooij et al examined the occurrence of victimisation in a Dutch out-patient sample of people with severe mental illness, which identified housemates as the most common perpetrators of victimisation (21%), followed by neighbours, with a prevalence of 16%;^31^ although we had no information on the identity of perpetrators in this study, it is possible that neighbourhood patterns we observed were explained partly by an association between neighbourhood crime and the prevalence of perpetrators with propensity to be violent in each neighbourhood.

In the general population, routine activities theory has been proposed to explain observed neighbourhood variation in the occurrence of physical victimisation. This model suggests that physical victimisation occurs as a result of perpetrators and vulnerable would-be victims coming together in spaces that are poorly supervised, and where perpetrators may perceive there to be a lower risk of getting caught.^32^ The association we reported for neighbourhood fragmentation persisted after taking account of neighbourhood crime, suggesting that social networks and access to support could be relevant in protecting individuals from physical victimisation. More fragmented neighbourhoods may reflect weaker community structures allowing residents to live safely. It may be more difficult in fragmented neighbourhoods for people with existing vulnerabilities to access support that may protect against experiencing violence. It is also possible that other area characteristics, not measured in the current study, could account for the associations we observed. For example, research suggests geographic variation exists in gender norms, beliefs which condone domestic violence,^33–35^ and stigmatising attitudes towards people with mental illness;^36,37^ neighbourhood patterns in violence towards people with mental illness observed in this study could reflect regional differences in attitudes. Material deprivation in neighbourhoods is associated with the experience of violence, across a range of indicators, including intimate partner violence,^38^ homicide^39^ and use of hospital care for assault.^40^ Our findings confirm a relationship between neighbourhood deprivation and victimisation in people with mental disorder.

### Strengths and limitations

Few studies to our knowledge have employed more than one method to identify or confirm physical victimisation in people with mental illness. The measurement of violence, and the possible role of gender in shaping measurement accuracy for different forms of violence, is complex,^41,42^ and may call for a broad range of measurement approaches. We used NLP to identify cases, and investigated data from administrative linkage of mental health records data with acute medical admissions to confirm our results. In this study, we were able to demonstrate similarities between an NLP-derived physical victimisation measure based on clinical text, and diagnoses from acute medical in-patient admissions for the same population.

However, our focus on any clinical recording of physical victimisation in electronic health records did not distinguish between violence experienced in childhood, adulthood, or distinguish among different settings and perpetrators for violence (including identifying partners as perpetrators). Physical violence can be accompanied by other types of violence and abuse, including psychological coercion and control, financial abuse, and sexual exploitation/abuse, which were not directly assessed in this study, and may have a substantial impact on health outcomes.^43^ Residual confounding of our final estimates is likely, for example by family or household characteristics. The temporal relationship between physical victimisation and onset and occurrence of mental illness could not be evaluated. Given that our study focused on neighbourhood associations, the absence of information on where instances of physical victimisation took place is also a limitation. Individuals included in this study may have been residing in areas very different to those where instances of physical victimisation actually took place, which is also a limitation to the analysis.

Both NLP and hospital admission data on physical assault used in this study likely under-ascertained the occurrence of the outcome. We found a low prevalence of physical victimisation in comparison with other studies employing self-report.^43,44^ The NLP algorithm was developed to ascertain clearly worded instances of physical victimisation in the records, in order to minimise false positives, so this is likely to represent a subset of wider cases, accounting for the low overall prevalence of physical victimisation identified. Considering the primary findings, and given that the algorithm was developed using machine learning to ascertain clinically identified instance of physical victimisation, it is unlikely that this misclassification was driven by neighbourhood characteristics of interest in this study. Other possible explanations for a low prevalence of recorded physical victimisation in these routine data, could be low levels of enquiry for, and disclosure of, violence in clinical settings, which have been consistently observed.44 Perpetration of violence has also been linked to neighbourhood characteristics in previous research, however, information on whether individuals were perpetrators of violence was not available in this study.

In line with previous epidemiological literature on neighbourhood characteristics, we identified small effects, and absolute risk differences which ranged from −0.38% to 1.36%. The study was based on a population drawn from mental health services and is therefore only generalisable to that group – the study did not directly investigate pathways to physical victimisation in the general population. Cases were defined through NLP of electronic health records, and these cases could be non-representative of the mental health service user population as a whole, for example, individuals with extensive records, such as those with more severe illnesses necessitating greater recording, could have been over-represented among the case group than the control group. Conscious or unconscious bias affecting the recording of information by clinicians cannot be excluded as an explanation for the patterns shown. Few previous studies have examined clinical recordings of physical victimisation, and this may have allowed investigation of a different population to those captured in national crime data and general population surveys, given that mental illness is associated with non-participation and non-reporting of crimes in research studies.^4^ On the other hand, our findings may not be generalisable to people using mental health services experiencing physical victimisation but where this is not identified and clinically recorded by mental health professionals.

### Implications

Physical violence towards people with mental illness is a fundamental health and social disparity. Where a person with mental illness lives may matter for their risk of experiencing physical violence. Strengthening social organisation and support in neighbourhoods could have an impact on the physical safety of people with mental illness, particularly women. As well as material deprivation, neighbourhood safety factors may be relevant for understanding physical violence towards people with mental illness. Public health strategies to improve the safety of community residents with mental illness could benefit from being gender sensitive. Incorporating information on physical victimisation from a number of measurement sources may be helpful for future research.

## Data Availability

Researcher access to data used in this study is available on application to the SLaM BRC Case Register Oversight Committee.

## Supplementary material

For supplementary material accompanying this paper visit http://dx.doi.org/10.1192/bjo.2020.52. click here to view supplementary material
