# Supplementary material for: The association between neighbourhood characteristics and physical victimisation in men and women with mental disorders
Source: BJPsych Open. 2020 Jul 16;6(4):e73. doi: 10.1192/bjo.2020.52 (PMC7443921; doi:10.1192/bjo.2020.52)
Supplement: Supplementary file 1 [file S2056472420000526sup001.zip › S2056472420000526sup001/RECORD Checklist VVNLPCRISv0.018.docx]

**The RECORD statement – checklist of items, extended from the STROBE statement, that should be reported in observational studies using routinely collected health data.**

|  | **Item No.** | **STROBE items** | **Location in manuscript where items are reported** | **RECORD items** | **Location in manuscript where items are reported** |
| --- | --- | --- | --- | --- | --- |
| **Title and abstract** | | | | | |
|  | 1 | (a) Indicate the study’s design with a commonly used term in the title or the abstract (b) Provide in the abstract an informative and balanced summary of what was done and what was found | Page 1 | RECORD 1.1: The type of data used should be specified in the title or abstract. When possible, the name of the databases used should be included.  RECORD 1.2: If applicable, the geographic region and timeframe within which the study took place should be reported in the title or abstract.  RECORD 1.3: If linkage between databases was conducted for the study, this should be clearly stated in the title or abstract. | Abstract: Page 1  Abstract: Page 1  Abstract : Page 1 |
| **Introduction** | | | | | |
| Background rationale | 2 | Explain the scientific background and rationale for the investigation being reported | Introduction: Page 2 |  |  |
| Objectives | 3 | State specific objectives, including any prespecified hypotheses | Introduction: Page 2 |  |  |
| **Methods** | | | | | |
| Study Design | 4 | Present key elements of study design early in the paper | First sentence of data source section, page 3. |  |  |
| Setting | 5 | Describe the setting, locations, and relevant dates, including periods of recruitment, exposure, follow-up, and data collection | Data source section, page 3. |  |  |
| Participants | 6 | *(a) Cohort study* - Give the eligibility criteria, and the sources and methods of selection of participants. Describe methods of follow-up  *Case-control study* - Give the eligibility criteria, and the sources and methods of case ascertainment and control selection. Give the rationale for the choice of cases and controls  *Cross-sectional study* - Give the eligibility criteria, and the sources and methods of selection of participants  *(b) Cohort study* - For matched studies, give matching criteria and number of exposed and unexposed  *Case-control study* - For matched studies, give matching criteria and the number of controls per case |  | RECORD 6.1: The methods of study population selection (such as codes or algorithms used to identify subjects) should be listed in detail. If this is not possible, an explanation should be provided.  RECORD 6.2: Any validation studies of the codes or algorithms used to select the population should be referenced. If validation was conducted for this study and not published elsewhere, detailed methods and results should be provided.  RECORD 6.3: If the study involved linkage of databases, consider use of a flow diagram or other graphical display to demonstrate the data linkage process, including the number of individuals with linked data at each stage. | Described in the section “identification of physical victimisation cases and controls”  Performance data on the NLP application for the definition of cases are described in the section entitled “*Development of a natural language processing (NLP) tool to identify cases and controls*”, on page 3.  Figure 1 demonstrates linkages included in the study. |
| Variables | 7 | Clearly define all outcomes, exposures, predictors, potential confounders, and effect modifiers. Give diagnostic criteria, if applicable. |  | RECORD 7.1: A complete list of codes and algorithms used to classify exposures, outcomes, confounders, and effect modifiers should be provided. If these cannot be reported, an explanation should be provided. | Variables are described in sections of the methods entitled: Neighbourhood characteristics and Other measurements. |
| Data sources/ measurement | 8 | For each variable of interest, give sources of data and details of methods of assessment (measurement).  Describe comparability of assessment methods if there is more than one group |  |  | Described in Data source, development of a natural language processing (NLP) tool to identify cases and controls, and neighbourhood characteristics sections. |
| Bias | 9 | Describe any efforts to address potential sources of bias |  |  | Efforts to address confounding bias is reported in the second paragraph of the analysis section(page 4), and in the results(page 5). Measurement bias of case-control status is examined using HES linkage, described in “identification of physical victimisation cases and controls”(page 3), and the prevalence of HES-derived physical assault is described in table 1. This is further examined in the discussion(page 7). Missing data: Selection bias introduced by missing data is examined in table S1, by assessing the association of interest in sub-populations with missing data on each covariate, and discussed in the section “missing data”, page 6. |
| Study size | 10 | Explain how the study size was arrived at |  |  | Decision on 10:1 matching described on page 3. |
| Quantitative variables | 11 | Explain how quantitative variables were handled in the analyses. If applicable, describe which groupings were chosen, and why |  |  | Described on page 4 and 5. |
| Statistical methods | 12 | (a) Describe all statistical methods, including those used to control for confounding  (b) Describe any methods used to examine subgroups and interactions  (c) Explain how missing data were addressed  (d) *Cohort study* - If applicable, explain how loss to follow-up was addressed  *Case-control study* - If applicable, explain how matching of cases and controls was addressed  *Cross-sectional study* - If applicable, describe analytical methods taking account of sampling strategy  (e) Describe any sensitivity analyses |  |  | 1. Covariate selection is described and referenced in the second paragraph of the analysis section, on pages 4 and 5. 2. Assessment of interaction and post-estimation fitted estimates is described in the second paragraph of the results, page 5. 3. Missing data handling is described in the final paragraph of the methods. 4. Matching and the derivation of the control series is described in “identification of physical victimisation cases and controls”, on page 3. |
| Data access and cleaning methods |  | .. |  | RECORD 12.1: Authors should describe the extent to which the investigators had access to the database population used to create the study population.  RECORD 12.2: Authors should provide information on the data cleaning methods used in the study. | Described in data source section, page 3.  Described in the Methods. |
| Linkage |  | .. |  | RECORD 12.3: State whether the study included person-level, institutional-level, or other data linkage across two or more databases. The methods of linkage and methods of linkage quality evaluation should be provided. | Described in the Data source section. |
| **Results** | | | | | |
| Participants | 13 | (a) Report the numbers of individuals at each stage of the study (*e.g.*, numbers potentially eligible, examined for eligibility, confirmed eligible, included in the study, completing follow-up, and analysed)  (b) Give reasons for non-participation at each stage.  (c) Consider use of a flow diagram |  | RECORD 13.1: Describe in detail the selection of the persons included in the study (*i.e.,* study population selection) including filtering based on data quality, data availability and linkage. The selection of included persons can be described in the text and/or by means of the study flow diagram. | Described in “identification of physical victimisation cases and controls”, page 3, and in the Figure 1. |
| Descriptive data | 14 | (a) Give characteristics of study participants (*e.g.*, demographic, clinical, social) and information on exposures and potential confounders  (b) Indicate the number of participants with missing data for each variable of interest  (c) *Cohort study* - summarise follow-up time (*e.g.*, average and total amount) |  |  | 1. A. Described in Table 1 and in the “Descriptive results” section of the results. 2. B. Described in the “missing data” section of the results. |
| Outcome data | 15 | *Cohort study* - Report numbers of outcome events or summary measures over time  *Case-control study* - Report numbers in each exposure category, or summary measures of exposure  *Cross-sectional study* - Report numbers of outcome events or summary measures |  |  | Described in table 1. |
| Main results | 16 | (a) Give unadjusted estimates and, if applicable, confounder-adjusted estimates and their precision (e.g., 95% confidence interval). Make clear which confounders were adjusted for and why they were included  (b) Report category boundaries when continuous variables were categorized  (c) If relevant, consider translating estimates of relative risk into absolute risk for a meaningful time period |  |  | 1. described in the results section, and covariate selection described in the analysis section of the methods. 2. Category boundaries for neighbourhood variables are described on page 4, and for other variables in the section “other variables”, page 4. |
| Other analyses | 17 | Report other analyses done—e.g., analyses of subgroups and interactions, and sensitivity analyses |  |  | Interaction tests are reported in table 5 and described on page 5. |
| **Discussion** | | | | | |
| Key results | 18 | Summarise key results with reference to study objectives |  |  | In “summary of findings” section, on page 6. |
| Limitations | 19 | Discuss limitations of the study, taking into account sources of potential bias or imprecision. Discuss both direction and magnitude of any potential bias |  | RECORD 19.1: Discuss the implications of using data that were not created or collected to answer the specific research question(s). Include discussion of misclassification bias, unmeasured confounding, missing data, and changing eligibility over time, as they pertain to the study being reported. | Described on pages 6 and 7, in the “strengths and limitations” section. |
| Interpretation | 20 | Give a cautious overall interpretation of results considering objectives, limitations, multiplicity of analyses, results from similar studies, and other relevant evidence |  |  | In discussion pages 6 and 7. |
| Generalisability | 21 | Discuss the generalisability (external validity) of the study results |  |  | Page 7. |
| **Other Information** | | | | | |
| Funding | 22 | Give the source of funding and the role of the funders for the present study and, if applicable, for the original study on which the present article is based |  |  | Page 9. |
| Accessibility of protocol, raw data, and programming code |  | .. |  | RECORD 22.1: Authors should provide information on how to access any supplemental information such as the study protocol, raw data, or programming code. | Page 5. |

*Reference: Benchimol EI, Smeeth L, Guttmann A, Harron K, Moher D, Petersen I, Sørensen HT, von Elm E, Langan SM, the RECORD Working Committee. The REporting of studies Conducted using Observational Routinely-collected health Data (RECORD) Statement. *PLoS Medicine* 2015; in press.

*Checklist is protected under Creative Commons Attribution ([CC BY](http://creativecommons.org/licenses/by/4.0/)) license.
