# Supplementary material for: The association between neighbourhood characteristics and physical victimisation in men and women with mental disorders
Source: BJPsych Open. 2020 Jul 16;6(4):e73. doi: 10.1192/bjo.2020.52 (PMC7443921; doi:10.1192/bjo.2020.52)
Supplement: Supplementary file 1 [file S2056472420000526sup001.zip › S2056472420000526sup001/UnmarkedMarkedSupplementary Tablesv0.018.doc - Recovered - Compatibility Mode.docx]

**Table S1. Specifications for free text searching application for physical victimisation in CRIS.**

*Keywords used in the first extraction:*

assault

hit

beaten up

stabbing

stabbed

knifed

punch

kick

beat

struck

victim

aggress

mugged

battered

strangle

throttle

scratch

bitten

*Phrases to improve specificity (and reduce efficiency) added before second round of annotations:*

was assaulted by

was hit by

was strangled by

Refinements to implement to search terms made after second round of annotations completed:

“Zzzz was [term]* by “

With these terms:

assault

hit

beaten up

stabbing

stabbed

knifed

punch

kick

beat

physical* victim*

aggress

mugged

battered

strangle

throttle

scratch

bitten

2. Excluding instances where “drive”, “car”, “truck”, “road”, “accident”, “vehicle”, “tram”, “bus”, “train”, or “vehicle” occurs up to three words after the phrase “ZZZZ was hit by..”.

3. Excluding instances where “bitten by” is followed within three words by ”mosquitoes” “spider” “something” “dog” “cat” “animal” “tics” “flea” or “fly”.

**Examples of instances of physical victimisation in clinical text identified by the NLP application developed in this study**

Randomly selected text fragments identified as containing physical victimisation by the natural language processing application developed for this study. “ZZZZ” is an automatically generated identifier for the patient’s name.

1. "ZZZZZ was assaulted by a shopkeeper who tried to strangle him resulting in bleeding through the nose."*
2. "ZZZZ said she regrets leaving her house even though she was assaulted by her husband."
3. "ZZZZ was assaulted by two youths at…".
4. “….two separate incidents at work where ZZZZ was assaulted by the same person at work."
5. "…he was beaten up by thughs outside a pub."
6. “...a reported incident where ZZZZ was beaten by his father."
7. "ZZZZZ said she was assaulted by 2 family members."
8. "he reports gang members managed to enter his flat and beat him and 'had an axe' with them"
9. "States that her son beat her up after she asked him and his friend to leave her house due to them drinking there."
10. "Patient was assaulted by another patient…"

| **Table S2. ICD-10 diagnostic codes used to classify physical victimisation in Hospital Episode Statistics (HES) data for this study.** | |
| --- | --- |
| **ICD-10 Code** | **Assault by:** |
| X85 | Drugs, medicaments and biological substances |
| X86 | Corrosive substance |
| X87 | Pesticides |
| X88 | Gases and vapours |
| X89 | Other specified chemicals and noxious substances |
| X90 | Unspecified chemical or noxious substance |
| X90 | Unspecified chemical or noxious substance |
| X91 | Hanging, strangulation and suffocation |
| X92 | Drowning and submersion |
| X93 | Handgun discharge |
| X94 | Rifle, shotgun and larger firearm discharge |
| X95 | Other and unspecified firearm discharge |
| X96 | Explosive material |
| X97 | Smoke, fire and flames |
| X98 | Steam, hot vapours and hot objects |
| X99 | Sharp object |
| Y00 | Blunt object |
| Y01 | Pushing from high place |
| Y02 | Pushing or placing victim before moving object |
| Y03 | Crashing of motor vehicle |
| Y04 | Bodily force |
| Y08 | Other specified means |
| Y09 | Unspecified means |

| **Table S3. Proportion of cases with medical admissions for physical victimisation within categories of each covariates.** | | | | | |
| --- | --- | --- | --- | --- | --- |
|  | No admission for physical victimisation | Admission for physical victimisation | Row total | Chi-squared | P value |
| Individual characteristics | |  |  |  |  |
| Age group |  |  |  |  |  |
| 0-15 | 1,227 | 66 | 1,293 |  |  |
| (%) | 94.9 | 5.1 | 100 |  |  |
| 16-24 | 1,374 | 160 | 1,534 |  |  |
| (%) | 89.57 | 10.43 | 100 |  |  |
| 25-35 | 1,616 | 180 | 1,796 |  |  |
| (%) | 89.98 | 10.02 | 100 |  |  |
| 36-50 | 1,533 | 176 | 1,709 |  |  |
| (%) | 89.7 | 10.3 | 100 |  |  |
| 51- | 823 | 55 | 878 | 42.39 | <0.001 |
| (%) | 93.74 | 6.26 | 100 |  |  |
| Missing | 3 | 0 | 3 |  |  |
| (%) | 100 | 0 | 100 |  |  |
| Gender |  |  |  |  |  |
| Female | 2,873 | 163 | 3,036 |  |  |
| (%) | 94.63 | 5.37 | 100 |  |  |
| Male | 3,703 | 474 | 4,177 | 78.06 | <0.001 |
| (%) | 88.65 | 11.35 | 100 |  |  |
| Ethnic group | |  |  |  |  |
| White | 3,372 | 350 | 3,722 |  |  |
| (%) | 91 | 9.4 | 100 |  |  |
| Mixed | 312 | 25 | 337 |  |  |
| (%) | 92.58 | 7.42 | 100 |  |  |
| Asian | 273 | 23 | 296 |  |  |
| (%) | 92.23 | 7.77 | 100 |  |  |
| Black | 1,975 | 187 | 2,162 |  |  |
| (%) | 91.35 | 8.65 | 100 |  |  |
| Other | 389 | 27 | 416 | 5.69 | 0.338 |
| (%) | 93.51 | 6.49 | 100 |  |  |
| Missing | 255 | 25 | 280 |  |  |
| (%) | 91.07 | 8.93 | 100 |  |  |
| Marital status | |  |  |  |  |
| Single | 4,434 | 472 | 4,906 |  |  |
| (%) | 90.38 | 9.62 | 100 |  |  |
| Married/  cohabiting | 711 | 53 | 764 |  |  |
| (%) | 93.06 | 6.94 | 100 |  |  |
| Divorced/  separated | 522 | 49 | 571 |  |  |
| (%) | 91.42 | 8.58 | 100 |  |  |
| Widowed | 179 | 11 | 190 | 14.05 | 0.007 |
| (%) | 94.21 | 5.79 | 100 |  |  |
| Missing | 730 | 52 | 782 |  |  |
| (%) | 93.35 | 6.65 | 100 |  |  |
| Primary diagnosis | |  |  |  |  |
| F0-9 | 204 | 18 | 222 |  |  |
| (%) | 91.89 | 8.11 | 100 |  |  |
| F10-19 | 424 | 99 | 523 |  |  |
| (%) | 81.07 | 18.93 | 100 |  |  |
| F20-29 | 1,363 | 109 | 1,472 |  |  |
| (%) | 92.6 | 7.4 | 100 |  |  |
| F30-39 | 1,104 | 73 | 1,177 |  |  |
| (%) | 93.8 | 6.2 | 100 |  |  |
| F40-49 | 570 | 49 | 619 |  |  |
| (%) | 92.08 | 7.92 | 100 |  |  |
| F50-59 | 64 | 1 | 65 |  |  |
| (%) | 98.46 | 1.54 | 100 |  |  |
| F60-69 | 213 | 24 | 237 |  |  |
| (%) | 89.87 | 10.13 | 100 |  |  |
| F70-79 | 148 | 8 | 156 |  |  |
| (%) | 94.87 | 5.13 | 100 |  |  |
| F80-89 | 143 | 6 | 149 |  |  |
| (%) | 95.97 | 4.03 | 100 |  |  |
| F90-98 | 371 | 32 | 403 |  |  |
| (%) | 92.06 | 7.94 | 100 |  |  |
| F99 | 689 | 71 | 760 |  |  |
| (%) | 90.66 | 9.34 | 100 |  |  |
| No axis I diagnosis | 317 | 21 | 338 |  |  |
| (%) | 93.79 | 6.21 | 100 |  |  |
| G | 14 | 0 | 14 |  |  |
| (%) | 100 | 0 | 100 |  |  |
| A-E, H-Q | 10 | 2 | 12 |  |  |
| (%) | 83.33 | 16.67 | 100 |  |  |
| R | 10 | 1 | 11 |  |  |
| (%) | 90.91 | 9.09 | 100 |  |  |
| S-Y | 11 | 1 | 12 |  |  |
| (%) | 91.67 | 8.33 | 100 |  |  |
| Z | 748 | 102 | 850 | 109.53 | <0.001 |
| (%) | 88 | 12 | 100 |  |  |
| Missing | 173 | 20 | 193 |  |  |
| (%) | 89.64 | 10.36 | 100 |  |  |
| Comorbid drug/alcohol | |  |  |  |  |
| No | 6,490 | 621 | 7,111 |  |  |
| (%) | 91.27 | 8.73 | 100 |  |  |
| Yes | 86 | 16 | 102 | 6.04 | 0.014 |
| (%) | 84.31 | 15.69 | 100 |  |  |
| Neighbourhood characteristics | | |  |  |  |
| Neighbourhood crime | |  |  |  |  |
| Higher | 2,689 | 250 | 2,939 |  |  |
| (%) | 91.49 | 8.51 | 100 |  |  |
| Lower | 3,501 | 315 | 3,816 | 0.1371 | 0.711 |
| (%) | 91.75 | 8.25 | 100 |  |  |
| Missing | 386 | 72 | 458 |  |  |
| (%) | 84.28 | 15.72 | 100 |  |  |
| Neighbourhood fragmentation | | |  |  |  |
| Higher | 2,803 | 258 | 3,061 |  |  |
| (%) | 91.57 | 8.43 | 100 |  |  |
| Lower | 3,010 | 279 | 3,289 | 0.006 | 0.938 |
| (%) | 91.52 | 8.48 | 100 |  |  |
| Missing | 763 | 100 | 863 |  |  |
| (%) | 88.41 | 11.59 | 100 |  |  |
| Neighbourhood income deprivation | | | | | |
| Higher | 2,654 | 215 | 2,869 |  |  |
| (%) | 92.51 | 7.49 | 100 |  |  |
| Lower | 3,536 | 350 | 3,886 | 4.9281 | 0.026 |
| (%) | 90.99 | 9.01 | 100 |  |  |
| Missing | 386 | 72 | 458 |  |  |
| (%) | 84.28 | 15.72 | 100 |  |  |
| Neighbourhood population density | | | | | |
| Higher | 2731 | 232 | 2963 |  |  |
| (%) | 92.17 | 7.83 | 100 |  |  |
| Lower | 3465 | 333 | 3798 |  |  |
| (%) | 91.23 | 8.77 | 100 | 1.91 | 0.167 |
| Missing | 380 | 72 | 452 |  |  |
| (%) | 84.07 | 15.93 | 100 |  |  |
| Total | 6,576 | 637 | 7,213 |  |  |
| (%) | 91.17 | 8.83 | 100 |  |  |

| **Table S4. Pairwise correlation matrix for neighbourhood characteristics examined in this study.** | | | | |
| --- | --- | --- | --- | --- |
|  | Neighbourhood fragmentation | Neighbourhood crime | Neighbourhood income deprivation | Neighbourhood population density |
| Neighbourhood fragmentation | 1 |  |  |  |
| Neighbourhood crime | 0.26 | 1 |  |  |
| Neighbourhood income deprivation | 0.07 | 0.43 | 1 |  |
| Neighbourhood population density | 0.32 | 0.25 | 0.35 | 1 |

| **Table S5. Comparing bivariable associations, in the form of the odds ratios(ORs) with covariates between natural language processing-derived cases (the method employed in this study) and cases meeting both the natural language processing and HES definitions.** | | | | | | |
| --- | --- | --- | --- | --- | --- | --- |
|  | NLP case (n=7213) | | | NLP case confirmed in HES (n=637) | | |
|  | OR | Lower 95% CI | Upper 95% CI | OR | Lower 95% CI | Upper 95% CI |
| Neighbourhood characteristics | |  |  |  |  |  |
| Neighbourhood fragmentation | 1.09 | 1.05 | 1.12 | 1.08 | 0.97 | 1.20 |
| Neighbourhood crime | 1.29 | 1.24 | 1.34 | 1.30 | 1.16 | 1.46 |
| Neighbourhood income deprivation | 1.25 | 1.21 | 1.29 | 1.40 | 1.28 | 1.53 |
| Neighbourhood population density | 1.13 | 1.10 | 1.17 | 1.13 | 1.09 | 1.17 |
| Individual characteristics | |  |  |  |  |  |
| Age group |  |  |  |  |  |  |
| 0-15 | 1.00 |  |  | 1.00 |  |  |
| 16-24 | 1.23 | 1.11 | 1.35 | 2.14 | 1.56 | 2.94 |
| 25-35 | 1.04 | 0.95 | 1.14 | 1.62 | 1.19 | 2.22 |
| 36-50 | 0.94 | 0.85 | 1.03 | 1.54 | 1.12 | 2.10 |
| 51+ | 0.38 | 0.35 | 0.42 | 0.40 | 0.27 | 0.60 |
| Gender |  |  |  |  |  |  |
| Female | 1.00 |  |  | 1.00 |  |  |
| Male | 1.41 | 1.34 | 1.49 | 1.41 | 1.34 | 1.49 |
| Primary diagnosis | |  |  |  |  |  |
| F0-9 | 1.00 |  |  | 1.00 |  |  |
| F10-19 | 1.78 | 1.50 | 2.12 | 3.65 | 2.16 | 6.14 |
| F20-29 | 9.02 | 7.70 | 10.57 | 7.79 | 4.68 | 12.95 |
| F30-39 | 2.98 | 2.55 | 3.48 | 2.11 | 1.25 | 3.54 |
| F40-49 | 1.94 | 1.64 | 2.29 | 1.74 | 0.98 | 3.09 |
| F50-59 | 0.73 | 0.54 | 0.99 | 0.15 | 0.02 | 1.09 |
| F60-69 | 5.98 | 4.84 | 7.38 | 7.27 | 3.82 | 13.83 |
| F70-79 | 5.33 | 4.18 | 6.80 | 3.93 | 1.69 | 9.15 |
| F80-89 | 2.71 | 2.12 | 3.47 | 1.71 | 0.67 | 4.34 |
| F90-98 | 2.86 | 2.37 | 3.47 | 2.77 | 1.47 | 5.19 |
| F99 | 1.73 | 1.46 | 2.05 | 2.03 | 1.21 | 3.40 |
| No diagnosis | 2.53 | 2.06 | 3.10 | 2.30 | 1.18 | 4.48 |
| G | 1.62 | 0.93 | 2.84 | - | - | - |
| A-E | 1.57 | 0.86 | 2.86 | 3.12 | 0.71 | 13.78 |
| R | 0.73 | 0.39 | 1.35 | 0.77 | 0.10 | 5.78 |
| S-Y | 2.91 | 1.42 | 5.97 | 3.92 | 0.51 | 30.25 |
| Z | 1.70 | 1.44 | 2.01 | 2.26 | 1.34 | 3.82 |
| Any comorbid diagnosis of drug or alcohol use disorder | |  |  |  |  |  |
| No | 1.00 |  |  | 1.00 |  |  |
| Yes | 2.16 | 1.74 | 2.68 | 3.57 | 2.11 | 6.05 |
| Marital status |  |  |  |  |  |  |
| Single | 1.00 |  |  | 1.00 |  |  |
| Married/cohabiting/civil partnership | 0.48 | 0.45 | 0.53 | 0.37 | 0.28 | 0.50 |
| Divorced/separated | 0.87 | 0.79 | 0.96 | 0.82 | 0.60 | 1.14 |
| Widowed | 0.31 | 0.27 | 0.37 | 0.19 | 0.10 | 0.36 |
| Ethnic group |  |  |  |  |  |  |
| White | 1.00 |  |  | 1.00 |  |  |
| Mixed | 1.93 | 1.69 | 2.20 | 1.69 | 1.07 | 2.65 |
| Asian | 1.13 | 0.98 | 1.29 | 0.93 | 0.59 | 1.47 |
| Black | 2.12 | 1.98 | 2.27 | 2.03 | 1.66 | 2.47 |
| Other | 0.93 | 0.83 | 1.05 | 0.76 | 0.50 | 1.15 |

| Table S6. Multivariable models for the association between neighbourhood fragmentation and disorder on physical victimisation in people using mental health care, all based on 44475 records with complete data on the modelled variables. | | | | | | | | | | | | | | | | | | | | | |
| --- | --- | --- | --- | --- | --- | --- | --- | --- | --- | --- | --- | --- | --- | --- | --- | --- | --- | --- | --- | --- | --- |
|  | Model with neighbourhood characteristics entered separately and unadjusted | | | Neighbourhood fragmentation with individual level covariates only | | | Neighbourhood crime with individual level covariates only | | | Neighbourhood income deprivation with individual level covariates only | | | Neighbourhood population density with individual level covariates only | | | Model with neighbourhood characteristics only (mutually adjusted) only | | | Final model, including all neighbourhood and individual level characteristics | | |
|  | OR | L95 | U95 | OR | L95 | U95 | OR | L95 | U95 | OR | L95 | U95 | OR | L95 | U95 | OR | L95 | U95 | OR | L95 | U95 |
| NEIGHBOURHOOD VARIABLES |  |  |  |  |  |  |  |  |  |  |  |  |  |  |  |  |  |  |  |  |  |
| **Neighbourhood fragmentation^a^** | **1.09** | **1.05** | **1.13** | **1.03** | **1.00** | **1.07** | **-** | **-** | **-** | **-** | **-** | **-** | **-** | **-** | **-** | **1.05** | **1.01** | **1.09** | **1.02** | **0.99** | **1.06** |
| **Neighbourhood crime** | **1.29** | **1.24** | **1.34** | **-** | **-** | **-** | **1.18** | **1.14** | **1.23** | **-** | **-** | **-** | **-** | **-** | **-** | **1.17** | **1.11** | **1.23** | **1.10** | **1.05** | **1.16** |
| **Neighbourhood income deprivation** | **1.25** | **1.21** | **1.29** | **-** | **-** | **-** | **-** | **-** | **-** | **1.15** | **1.11** | **1.19** | **-** | **-** | **-** | **1.18** | **1.13** | **1.23** | **1.12** | **1.07** | **1.16** |
| **Neighbourhood population density** | **1.13** | **1.10** | **1.17** | **-** | **-** | **-** | **-** | **-** | **-** | **-** | **-** | **-** | **1.05** | **1.02** | **1.09** | **1.03** | **0.99** | **1.07** | **0.98** | **0.94** | **1.01** |
| LSOA-level random effect |  |  |  | 0.02 | 0.01 | 0.06 | 0.02 | 0.01 | 0.06 | 0.02 | 0.01 | 0.06 | 0.03 | 0.01 | 0.06 | 0.04 | 0.02 | 0.08 | 0.01 | 0.00 | 0.07 |
| INDIVIDUAL VARIABLES |  |  |  |  |  |  |  |  |  |  |  |  |  |  |  |  |  |  |  |  |  |
| Age^b^ | **-** | **-** | **-** | 0.98 | 0.98 | 0.98 | 0.98 | 0.98 | 0.98 | 0.98 | 0.98 | 0.98 | 0.98 | 0.98 | 0.98 | **-** | **-** | **-** | 0.98 | 0.98 | 0.98 |
| Gender |  |  |  |  |  |  |  |  |  |  |  |  |  |  |  |  |  |  |  |  |  |
| Female | **-** | **-** | **-** | Ref |  |  | Ref |  |  | Ref |  |  | Ref |  |  | **-** | **-** | **-** | Ref |  |  |
| Male | **-** | **-** | **-** | 1.28 | 1.21 | 1.36 | 1.27 | 1.20 | 1.35 | 1.27 | 1.20 | 1.58 | 1.27 | 1.20 | 1.35 | **-** | **-** | **-** | 1.28 | 1.20 | 1.36 |
| Ethnic group |  |  |  |  |  |  |  |  |  |  |  |  |  |  |  |  |  |  |  |  |  |
| British | **-** | **-** | **-** | Ref |  |  | Ref |  |  | Ref |  |  | Ref |  |  | **-** | **-** | **-** | Ref |  |  |
| Mixed | **-** | **-** | **-** | 1.34 | 1.17 | 1.54 | 1.37 | 1.20 | 1.57 | 1.38 | 1.20 | 1.58 | 1.39 | 1.22 | 1.60 | **-** | **-** | **-** | 1.33 | 1.16 | 1.52 |
| Asian | **-** | **-** | **-** | 0.95 | 0.83 | 1.10 | 0.97 | 0.84 | 1.11 | 0.98 | 0.86 | 1.13 | 0.98 | 0.86 | 1.13 | **-** | **-** | **-** | 0.95 | 0.82 | 1.09 |
| Black | **-** | **-** | **-** | 1.41 | 1.31 | 1.51 | 1.44 | 1.34 | 1.55 | 1.44 | 1.34 | 1.55 | 1.48 | 1.37 | 1.59 | **-** | **-** | **-** | 1.37 | 1.27 | 1.47 |
| Other | **-** | **-** | **-** | 0.73 | 0.65 | 0.83 | 0.75 | 0.67 | 0.85 | 0.76 | 0.68 | 0.86 | 0.76 | 0.68 | 0.86 | **-** | **-** | **-** | 0.72 | 0.64 | 0.82 |
| Marital status |  |  |  |  |  |  |  |  |  |  |  |  |  |  |  |  |  |  |  |  |  |
| Single | **-** | **-** | **-** | Ref |  |  | Ref |  |  | Ref |  |  | Ref |  |  | **-** | **-** | **-** | Ref |  |  |
| Married/cohabiting | **-** | **-** | **-** | 0.77 | 0.70 | 0.85 | 0.78 | 0.71 | 0.85 | 0.78 | 0.71 | 0.85 | 0.78 | 0.71 | 0.85 | **-** | **-** | **-** | 0.78 | 0.71 | 0.85 |
| Divorced/separated | **-** | **-** | **-** | 1.16 | 1.04 | 1.30 | 1.17 | 1.05 | 1.30 | 1.16 | 1.04 | 1.29 | 1.17 | 1.05 | 1.30 | **-** | **-** | **-** | 1.15 | 1.03 | 1.29 |
| Widowed | **-** | **-** | **-** | 0.84 | 0.70 | 1.01 | 0.84 | 0.70 | 1.01 | 0.84 | 0.70 | 1.00 | 0.84 | 0.70 | 1.01 | **-** | **-** | **-** | 0.83 | 0.69 | 1.00 |
| Primary diagnosis |  |  |  |  |  |  |  |  |  |  |  |  |  |  |  |  |  |  |  |  |  |
| F0-9: Organic, including symptomatic, mental disorders | **-** | **-** | **-** | Ref |  |  | Ref |  |  | Ref |  |  | Ref |  |  | **-** | **-** | **-** | Ref |  |  |
| F10-19: Mental and behavioural disorders due to psychoactive substance use | **-** | **-** | **-** | 0.79 | 0.65 | 0.95 | 0.83 | 0.69 | 1.00 | 0.83 | 0.69 | 0.99 | 0.83 | 0.69 | 1.00 | **-** | **-** | **-** | 0.79 | 0.65 | 0.95 |
| F20-29: Schizophrenia, schizotypal and delusional disorders | **-** | **-** | **-** | 3.84 | 3.24 | 4.55 | 3.91 | 3.31 | 4.62 | 3.94 | 3.34 | 4.64 | 3.94 | 3.34 | 4.65 | **-** | **-** | **-** | 3.82 | 3.23 | 4.53 |
| F30-39: Mood (affective) disorders | **-** | **-** | **-** | 1.56 | 1.32 | 1.85 | 1.61 | 1.37 | 1.89 | 1.62 | 1.38 | 1.91 | 1.62 | 1.38 | 1.91 | **-** | **-** | **-** | 1.56 | 1.32 | 1.85 |
| F40-49: Neurotic, stress-related and somatoform disorders | **-** | **-** | **-** | 0.94 | 0.78 | 1.13 | 0.95 | 0.79 | 1.14 | 0.95 | 0.79 | 1.13 | 0.96 | 0.79 | 1.13 | **-** | **-** | **-** | 0.94 | 0.78 | 1.13 |
| F50-59: Behavioural syndromes associated with physiological disturbances and physical factors | **-** | **-** | **-** | 0.33 | 0.24 | 0.45 | 0.36 | 0.26 | 0.48 | 0.36 | 0.27 | 0.50 | 0.35 | 0.26 | 0.48 | **-** | **-** | **-** | 0.34 | 0.25 | 0.47 |
| F60-69: Disorders of adult personality and behaviour | **-** | **-** | **-** | 2.60 | 2.06 | 3.28 | 2.91 | 2.34 | 3.63 | 2.90 | 2.33 | 3.61 | 2.90 | 2.33 | 3.61 | **-** | **-** | **-** | 2.59 | 2.05 | 3.26 |
| F70-79: Mental retardation | **-** | **-** | **-** | 1.79 | 1.36 | 2.34 | 2.07 | 1.60 | 2.69 | 2.09 | 1.61 | 2.72 | 2.10 | 1.62 | 2.72 | **-** | **-** | **-** | 1.78 | 1.36 | 2.33 |
| F80-89: Disorders of psychological development | **-** | **-** | **-** | 0.68 | 0.51 | 0.90 | 0.76 | 0.58 | 1.00 | 0.76 | 0.58 | 0.99 | 0.76 | 0.58 | 0.99 | **-** | **-** | **-** | 0.68 | 0.51 | 0.91 |
| F90-98: Behavioural and emotional disorders with onset usually occurring in childhood and adolescence | **-** | **-** | **-** | 0.74 | 0.59 | 0.92 | 0.79 | 0.64 | 0.98 | 0.78 | 0.63 | 0.97 | 0.80 | 0.64 | 0.99 | **-** | **-** | **-** | 0.73 | 0.58 | 0.91 |
| F99: Unspecified mental disorder | **-** | **-** | **-** | 0.89 | 0.74 | 1.07 | 0.88 | 0.74 | 1.05 | 0.87 | 0.73 | 1.05 | 0.88 | 0.74 | 1.06 | **-** | **-** | **-** | 0.89 | 0.74 | 1.07 |
| No axis I diagnosis | **-** | **-** | **-** | 0.65 | 0.51 | 0.82 | 0.67 | 0.54 | 0.85 | 0.67 | 0.53 | 0.84 | 0.68 | 0.54 | 0.86 | **-** | **-** | **-** | 0.64 | 0.51 | 0.81 |
| G: Diseases of the nervous system | **-** | **-** | **-** | 1.40 | 0.74 | 2.64 | 1.23 | 0.70 | 2.16 | 1.18 | 0.67 | 2.07 | 1.19 | 0.68 | 2.08 | **-** | **-** | **-** | 1.42 | 0.75 | 2.69 |
| A-E, H-Q: Other illness codes | **-** | **-** | **-** | 0.85 | 0.46 | 1.56 | 0.79 | 0.43 | 1.46 | 0.79 | 0.43 | 1.46 | 0.80 | 0.43 | 1.47 | **-** | **-** | **-** | 0.83 | 0.45 | 1.54 |
| R: Symptoms, signs and abnormal clinical and laboratory findings, not elsewhere classified | **-** | **-** | **-** | 0.36 | 0.18 | 0.73 | 0.40 | 0.21 | 0.75 | 0.40 | 0.21 | 0.75 | 0.39 | 0.21 | 0.72 | **-** | **-** | **-** | 0.38 | 0.19 | 0.76 |
| S-Y: Injury, poisoning and external causes | **-** | **-** | **-** | 1.42 | 0.62 | 3.25 | 1.48 | 0.68 | 3.23 | 1.45 | 0.66 | 3.17 | 1.46 | 0.67 | 3.20 | **-** | **-** | **-** | 1.43 | 0.63 | 3.28 |
| Z: Factors influencing health status and contact with health services | **-** | **-** | **-** | 0.92 | 0.77 | 1.10 | 0.92 | 0.77 | 1.10 | 0.92 | 0.78 | 1.10 | 0.93 | 0.78 | 1.10 | **-** | **-** | **-** | 0.92 | 0.76 | 1.10 |
| Any comorbid diagnosis of drug or substance use disorder |  |  |  |  |  |  |  |  |  |  |  |  |  |  |  |  |  |  |  |  |  |
| No | **-** | **-** | **-** | Ref |  |  | Ref |  |  | Ref |  |  | Ref |  |  | **-** | **-** | **-** | Ref |  |  |
| Yes | **-** | **-** | **-** | 2.05 | 1.56 | 2.70 | 2.01 | 1.54 | 2.62 | 2.01 | 1.54 | 2.62 | 2.04 | 1.56 | 2.66 | **-** | **-** | **-** | 2.01 | 1.52 | 2.64 |
| 1. A neighbourhood fragmentation squared term was estimated at 0.97(95%CI:0.95,1.00) in the final model. 2. Age was modelled as a continuous term. | | | | | | | | | | | | | | | | | | | | | |

| **Table S7. Missing data on included variables in the study, by case-control status, and presenting the main associations among subgroups with missing data on each variable. Dashes(-) indicate comparisons with insufficient observations with which to estimate the associations.** | | | | | | |
| --- | --- | --- | --- | --- | --- | --- |
| Number of participants with missing data | Controls(%) | Cases(%) | Association with between physical victimisation and neighbourhood fragmentation within individuals with missing data on variable | Association with between physical victimisation and neighbourhood crime within individuals with missing data on variable | Association with between physical victimisation and neighbourhood income deprivation within individuals with missing data on variable | Association with between physical victimisation and neighbourhood population density within individuals with missing data on variable |
| Individual characteristics | | | | |  |  |
| Age | 70(0.1) | 3(0.04) | - | - | - | - |
| Gender | 20(0.03) | 0(0.00) | - | - | - | - |
| Ethnic group | 15622(21.66) | 280(3.88) | 1.03(0.91,1.18) | 1.11(0.95,1.29) | 1.17(1.03, 1.32) | 1.09(0.96, 1.23) |
| Marital status | 21476(29.77) | 786(10.84) | 1.17(1.07,1.28) | 0.98(0.90,1.06) | 1.22(1.13, 1.33) | 1.14(1.06, 1.23) |
| Primary diagnosis | 9106(12.62) | 193(2.68) | 1.20(0.98,1.45) | 1.14(0.98,1.34) | 1.14(0.98, 1.33) | 1.17(1.01, 1.34) |
| Comorbid diagnosis of drug or alcohol use disorders | 0(0.00) | 0(0.00) | - | - | - | - |
| Admission for physical victimisation | 0(0.00) | 0(0.00) | - | - | - | - |
| Neighbourhood characteristics | | | | |  |  |
| Neighbourhood fragmentation | 10830(15.00) | 863(11.96) | - | 1.07(0.97,1.17) | 1.05(0.96, 1.17) | 1.00(0.81, 1.23) |
| Neighbourhood crime | 4233(5.87) | 458(6.35) | - | - | - | - |
| Neighbourhood income deprivation | 4233(5.87) | 458(6.35) | - | - | - | - |
| Neighbourhood population density | 4146(5.75) | 452(6.26) | - | - | - | - |
