# Supplementary material for: The association between neighbourhood characteristics and physical victimisation in men and women with mental disorders
Source: BJPsych Open. 2020 Jul 16;6(4):e73. doi: 10.1192/bjo.2020.52 (PMC7443921; doi:10.1192/bjo.2020.52)
Supplement: Supplementary file 1 [file S2056472420000526sup001.zip › S2056472420000526sup001/MethodologicalSupplementv0.013.doc]

Methodological Supplement

*Development and evaluation of a natural language processing (NLP) tool to identify cases and controls*

Records in CRIS containing a lifetime history of physical victimisation were ascertained by a natural language processing (NLP) algorithm, which we developed for the purposes of this study. Natural language processing (“text-mining”) methods allow for automatic extraction of relevant textual information from clinical and other forms of data in electronic health records. The NLP software package TextHunter^30^ was used to extract documentation of physical victimisation from unstructured free text fields of clinical assessments and correspondence in the CRIS^31^, using similar protocols to other algorithms for ascertaining clinical information, published elsewhere^32^. Algorithm development was based on a “bag of words” model. An algorithm gazetteer was developed by two investigators (JJ and VB), listing keywords (see online supplementary table S1) to identify particular clinical text descriptions of physical victimisation. Search queries on CRIS were run and search results reviewed, allowing refinement of keywords and the inclusion of modifier words to make queries more precise. Having finalised the gazetteer, a random sample of 2200 sentences in CRIS containing gazetteer keywords and modifier words were then extracted and used as a basis to develop an NLP algorithm to identify specific descriptions of physical victimisation. Firstly, a human annotator (VB) classified the presence or absence of the construct in 498 of these sentences, to generate a reference data set for subsequent precision testing. A supervised machine learning approach was then used to identify sentences containing physical victimisation – in this step, a hundred further sentences were classified by a human annotator to generate a training data set, on which a support vector machine-learning algorithm was applied, in order to develop an NLP algorithm to automatically identify physical victimisation. The algorithm was iteratively tested against the reference data set of 498 text fragments to optimize precision and to reduce the probability of false-positive results, with a total of three iterations before satisfactory performance was reached. We defined the numbers of text fragments used for NLP algorithm development based on those used for previous NLP algorithms that have displayed adequate performance. Final precision (positive predictive value) and recall (sensitivity) statistics at a sentence level for the final algorithm, based on comparison with manual rating, were 0.92 and 0.98, respectively for these particular types of recorded victimisation. This suggested satisfactory performance of the algorithm at ascertaining clearly recorded clinical descriptions of physical victimisation in patient records (rather than the identification of all physical victimisation). Development of the NLP algorithm took place between 2015 and 2017.

*Other measurements*

Information on date of birth, date of referral to mental health services, gender, and marital status (classified into single, married/cohabiting, divorced/separated, and widowed) were taken from structured fields in CRIS, as close as possible to the referral date. Self-reported ethnicity was available in ethnic categories classified as per the 2011 UK Census, and collapsed into a white category (comprising individuals of white British, white mixed, and white other ethnicity), mixed (white and black Caribbean, white and black African, and white and Asian background), South Asian (comprising any other Asian background, Bangladeshi, Indian and Pakistani), black (black Caribbean, African, and any other black background) and Other (Chinese and any other ethnic group). Primary ICD-10 diagnosis^39^ was taken from a structured field for clinical diagnosis, and classified as follows: organic mental disorders (ICD-10 codes F0-9), mental and behavioural disorders due to psychoactive substance use (F10-19), schizophrenia, schizotypal and delusional disorders (F20-29), mood (affective) disorders (F30-39), neurotic, stress-related and somatoform disorders (F40-49), behavioural syndromes associated with physiological disturbances and physical factors (F50-59), disorders of adult personality and behaviour (F60-69), learning disability (F70-79), disorders of psychological development (F80-89), behavioural and emotional disorders with onset usually occurring in childhood and adolescence (F90-98), unspecified mental disorders(F99), no axis 1 diagnosis, diseases of the nervous system (G), other illness codes (A-E, H-Q), symptoms, signs and abnormal clinical and laboratory findings, not elsewhere classified (R), injury, poisoning and external causes (S-Y), and factors influencing health status and contact with health services (Z). Secondary ICD-10 diagnoses of alcohol and drug use disorders (F10-19) were also taken from a structured field in CRIS, and used to generate a binary variable for the presence of any comorbid alcohol or drug use disorders.

*Data management and sharing statement*

The study complied with the CRIS data security model^31^. The study protocol and analysis code are available from the corresponding author. The raw data is generated by the NHS and is only available to NHS staff (including those with honorary NHS contracts). Data access can be gained on request, subject to NHS access authorisation.
